# Decomposing Organic Molecules on Titanium with Vacuum Ultraviolet Light for Effective and Rapid Photofunctionalization

**DOI:** 10.3390/jfb14010011

**Published:** 2022-12-23

**Authors:** Toshikatsu Suzumura, Takanori Matsuura, Keiji Komatsu, Takahiro Ogawa

**Affiliations:** 1 Weintraub Center for Reconstructive Biotechnology, UCLA School of Dentistry, Los Angeles, CA 90095-1668, USA; 2 Division of Regenerative and Reconstructive Sciences, UCLA School of Dentistry, Los Angeles, CA 90095-1668, USA

**Keywords:** UV photofunctionalization, hydrocarbon, dental and orthopedic implants, biological aging of titanium

## Abstract

Ultraviolet (UV) photofunctionalization counteracts the biological aging of titanium to increase the bioactivity and osseointegration of titanium implants. However, UV photofunctionalization currently requires long treatment times of between 12 min and 48 h, precluding routine clinical use. Here, we tested the ability of a novel, xenon excimer lamp emitting 172 nm vacuum UV (VUV) to decompose organic molecules coated on titanium as a surrogate of photofunctionalization. Methylene blue as a model organic molecule was coated on grade 4 commercially pure titanium and treated with four UV light sources: (i) ultraviolet C (UVC), (ii) high-energy UVC (HUVC), (iii) proprietary UV (PUV), and (iv) VUV. After one minute of treatment, VUV decomposed 57% of methylene blue compared with 2%, 36%, and 42% for UVC, HUVC, and PUV, respectively. UV dose-dependency testing revealed maximal methylene blue decomposition with VUV within one minute. Equivalent decomposition was observed on grade 5 titanium alloy specimens, and placing titanium specimens in quartz ampoules did not compromise efficacy. Methylene blue was decomposed even on polymethyl methacrylate acrylic specimens at 20–25% lower efficiency than on titanium specimens, indicating a relatively small contribution of titanium dioxide-mediated photocatalytic decomposition to the total decomposition. Load-testing revealed that VUV maintained high efficacy of methylene blue decomposition regardless of the coating density, whereas other UV light sources showed low efficacy with thin coatings and plateauing efficacy with thicker coatings. This study provides foundational data on rapid and efficient VUV-mediated organic decomposition on titanium. In synergy with quartz ampoules used as containers, VUV has the potential to overcome current technical challenges hampering the clinical application of UV photofunctionalization.

## 1. Introduction

UV photofunctionalization—the treatment of titanium with UV light—is now an established approach to increase the osteoconductivity of titanium-based implant materials [1,2,3,4,5,6,7,8,9,10,11,12,13,14,15,16,17]. Despite demand and high expectation in dental and orthopedic practice, its clinical uptake has not matched its potential due to the need for long exposure times [4,18]. UV photofunctionalization was originally developed as a 48 h treatment [19,20,21,22], although this was later improved to 30-, 20-, and 12 min treatments [1,2,6,7,9,10,11,23,24,25,26,27,28]. Although theoretically possible to use in the clinic, current UV photofunctionalization protocols create unacceptable delay after preparation of the implant host site in bone. Consequently, treatment must be completed before the host bone site is prepared, risking a mismatch in the size of the host bone site and implant. Treating titanium implants ahead of time creates a storage problem [20,29,30,31,32], and there remains a need to further optimize UV photofunctionalization for clinical practice.

In addition to these practical problems, there remain knowledge gaps about the efficacy of UV photofunctionalization. While the biological and clinical effects of photofunctionalization have now been extensively studied [2,5,9,15,17,19,33,34,35,36,37,38,39,40,41,42,43,44,45,46,47,48,49,50,51,52,53,54,55], there is still uncertainty about its maximal effect and the optimal treatment time [37]. Specifically, there are still unanswered questions about the relationship between treatment time and efficacy and therefore what the shortest possible treatment time—which would facilitate clinical practice—is.

UV photofunctionalization can be regarded as the promotion of osteoconductivity and, technically, the decarbonization of titanium surfaces [19,20,22,23,25,26,33,41,42,56,57,58,59,60,61,62,63]. Titanium surfaces accumulate atmospheric hydrocarbons over time, which eventually cover 40–75% of the titanium surface [3,4,19,29,30,33,62,64,65,66]. These surface hydrocarbons decrease the wettability of titanium surfaces, i.e., the surfaces become hydrophobic [29,30,62]. Surface hydrocarbons and hydrophobicity together reduce the recruitment, attachment, and proliferation of osteogenic cells on titanium as well as blood flow and protein recruitment [32,67,68], thereby compromising bone-to-titanium integration, or osseointegration [18,20,29,45,62,69,70]. This time-dependent degradation of the surface properties and bioactivity of titanium is regarded as a form of “biological aging” [18,25,29,30,31,32]. UV photofunctionalization can reverse the biological aging of titanium and hence improve osteoconductivity [26,40,43,61,63,71,72,73,74,75,76,77,78,79,80,81].

UV light removes carbon-containing molecules via two mechanisms: direct decomposition (photolysis) and titanium dioxide-mediated decomposition (photocatalysis) [19,82,83,84,85,86,87,88,89,90,91,92,93,94,95,96,97]. The former is sub-categorized into photochemical and photophysical decomposition. Both UVA (315–380 nm wavelength) and UVC (200–280 nm) have been used for UV photofunctionalization, but their delivery to titanium surfaces is limited by their UV energy and permeability. UVC, which is a higher energy source, more effectively decomposes hydrocarbons but is quickly absorbed by the atmosphere and decays significantly before reaching the titanium surface [85,98,99,100,101]. By virtue of this lack of permeability, titanium implants must be UV treated after unpacking implant products from their sterile containers [1,2,6,7,8,9,10,11,28], which can introduce bacterial contamination. Current UV photon energy sources do not sufficiently shorten the treatment to improve clinical viability, and novel UV energy sources require further investigation.

Therefore, the objective of this study was to test the potential of a novel, xenon excimer lamp emitting 172 nm vacuum UV (VUV, wavelength < 200 nm) for UV photofunctionalization. VUV was compared with other UV light sources in its ability to decompose carbon-containing molecules, the most useful surrogate of photofunctionalization, using methylene blue as a model molecule. We hypothesized that the higher photon energy of VUV would more effectively mediate organic decomposition to reach maximal effect within a clinically acceptable timeframe. We also determined whether quartz is a viable material for containing titanium specimens for UV treatment so that implants can remain in their product packaging during UV treatment to preserve sterility.

## 2. Materials and Methods

### 2.1. Test Specimens and Surface Wettability Testing

**Figure 1:**
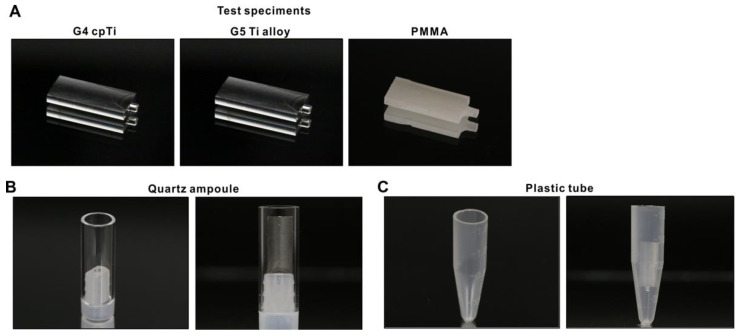
Test specimens and containers used in this study. (**A**) Test specimens made of different materials. (**B**) An ampoule made of synthetic quartz. A grade 4 titanium specimen placed in the quartz ampoule (right panel). (**C**) A laboratory-grade clear plastic tube. A grade 4 titanium specimen placed in the plastic tube (right panel).

Titanium test specimens in rectangular plate form (14 mm × 6 mm, 2 mm thickness) were machine-milled from commercially pure grade 4 titanium and grade 5 Ti-6Al-4V alloy (Figure 1A). Poly(methyl methacrylate) (PMMA) acrylic resin specimens were also fabricated. Surface morphology of these test specimens was examined by scanning electron microscopy (SEM; Nova 230 Nano SEM, FEI, Hillsboro, Oregon). The hydrophilicity/hydrophobicity of test surfaces with and without UV treatment were evaluated by measuring the contact angle of 3 µL of ddH_2_O.

### 2.2. Methylene Blue as a Model Organic Molecule and Specimen Containers

Methylene blue was used as a model organic molecule to decompose using UV light. Five µL of 0.07% stock solution was placed on test specimens, followed by drying in air for 12 h. Quartz ampoules in hollow cylinder form (10 mm diameter, 25 mm height, 1 mm thick) made from synthetic quartz glass (Figure 1B) and laboratory-grade 1.5 mL plastic tubes (Fisher Scientific, Pittsburgh, PA, USA) (Figure 1C) were prepared as specimen containers for UV treatment.

### 2.3. UV-Mediated Decomposition of Methylene Blue

Test specimens coated with methylene blue were placed in either quartz ampoules or plastic tubes and treated with four different UV light sources: (i) UVC from a commercially available low-pressure mercury lamp (1.2 mW/cm^2^; Iwasaki Electric, Tokyo, Japan); (ii) high-energy UVC (HUVC) from a commercially available UV device for dental implants (TheraBeam Affiny, Ushio, Tokyo, Japan); (iii) a commercially available UV device for dental implants with a proprietary protocol (PUV) (SuperOsseo, Ushio, Tokyo, Japan); and (iv) a xenon excimer lamp emitting 172 nm vacuum UV (VUV; ~60 mW/cm^2^) (DIO, Busan, Republic of Korea). Specimens were irradiated with UVC and VUV at a distance of 6 mm and HUVC and PUV following the manufacturers’ instructions for one minute except for dose-dependency experiments, where the exposure time was varied. Methylene blue remaining on the test specimen after UV treatment was dissolved in 800 µL ddH_2_O and quantified using a microplate reader at 650 nm (Synergy H1, BioTek Instruments, Winooski, VT, USA). Remaining methylene blue was calculated as a percentage relative to the total coated amount.

### 2.4. Statistical Analyses

Three test specimens were used for all methylene blue decomposition experiments. The effect of different UV light sources, test specimens, treatment times, and treatment conditions were compared by one-way ANOVA. Bonferroni’s test was used as a post hoc multiple comparison test where appropriate. *p*-values < 0.05 were considered statistically significant. Regression analysis was applied to determine the associations between remaining methylene blue, UV treatment time, a load-testing parameter, and surface hydrophilicity/hydrophobicity.

## 3. Results

### 3.1. Surface Morphology of Test Specimens

**Figure 2:**
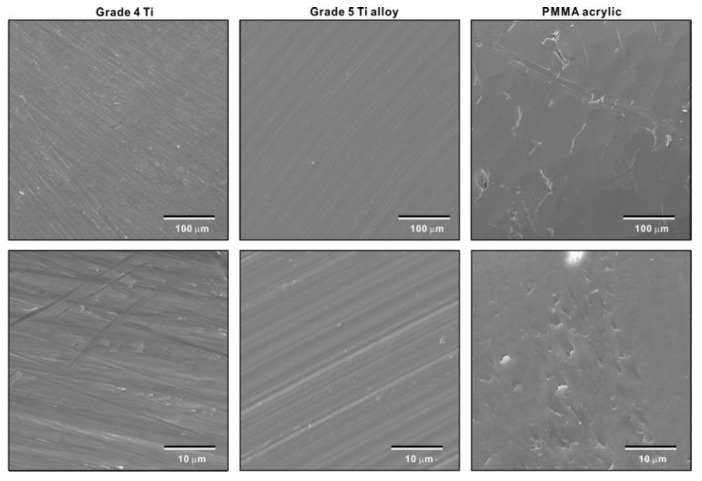
Surface morphology of test specimens. Low- and high-magnification SEM images are shown.

Low- and high-magnification SEM images of grade 4 titanium and grade 5 titanium alloy specimens showed a similar texture with line traces and minor irregularities made by machined turning (Figure 2). PMMA acrylic specimens showed a slight wavy morphology with scratches and irregularities made by milling.

### 3.2. Decomposition of Organic Molecules by Different UV Light Sources

**Figure 3:**
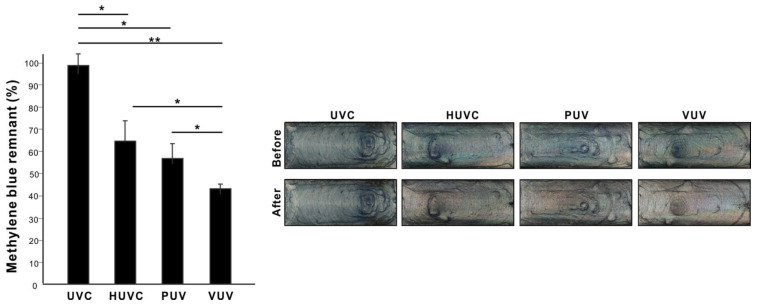
UV-mediated decomposition of methylene blue by various UV sources. Grade 4 titanium specimens coated with methylene blue were placed in quartz ampoules and treated with UV light for one minute. Four different UV light sources were used: (1) UVC from a low-pressure mercury lamp (UVC); (2) high-energy UVC (HUVC); (3) a UV device with a proprietary protocol (PUV); and 4) 172 nm vacuum UV (VUV). A histogram showing remnant methylene blue after UV treatment (%). Photographs of methylene blue-coated titanium specimens before and after UV treatment are also shown. * *p* < 0.05, ** *p* < 0.01.

We first compared the ability of VUV light to decompose organic molecules with UVC, HUVC, and PUV. Titanium specimens coated with methylene blue, used as a model carbon-containing molecule, were placed in quartz ampoules and treated with the UV light sources for one minute. There was significant variability in post-treatment residual methylene blue depending on the light source used, with UVC the highest and VUV the lowest (*p* < 0.01, ANOVA; Figure 3); VUV decomposed >55% of the coated methylene blue in one minute, while UVC only decomposed <5%. Representative photographs of the titanium specimens clearly illustrate the color changes before and after each UV treatment and between the different UV sources (Figure 3).

### 3.3. Identification of Maximum Efficacy of VUV

**Figure 4:**
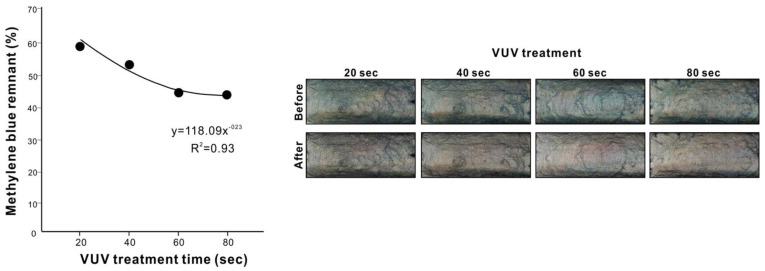
Dose-dependency and optimization of VUV-mediated organic decomposition. Methylene blue-coated grade 4 titanium specimens in quartz ampoules were treated with VUV for various treatment times from 20 to 80 s. The remaining methylene blue was quantified and plotted against UV treatment time and showed a highly fitted, negative exponential curve. Photographs of methylene blue-coated grade 4 titanium specimens before and after VUV treatment are shown.

After establishing that VUV was the most effective light source after only one minute of treatment, we next examined VUV exposure dose-dependency (20 to 80 s) to optimize the treatment time and maximize decomposition of methylene blue in a quartz ampoule. Residual methylene blue decreased with increasing time up to 60 s (*p* < 0.01) and then plateaued with longer treatments (Figure 4; no difference between 60- and 80-s treatments). The treatment time-residual methylene blue plot showed a near perfect fit to a negative exponential curve with a very high coefficient of determination (R^2^ = 0.93), confirming a limit to the dose-dependency with maximum decomposition at 60 s. Representative photographs of methylene blue-coated titanium specimens showed progressively less blue discoloration from 20 s to 60 s of VUV treatment (Figure 4).

### 3.4. Effect of Quarts Ampoules on Organic Decomposition

**Figure 5:**
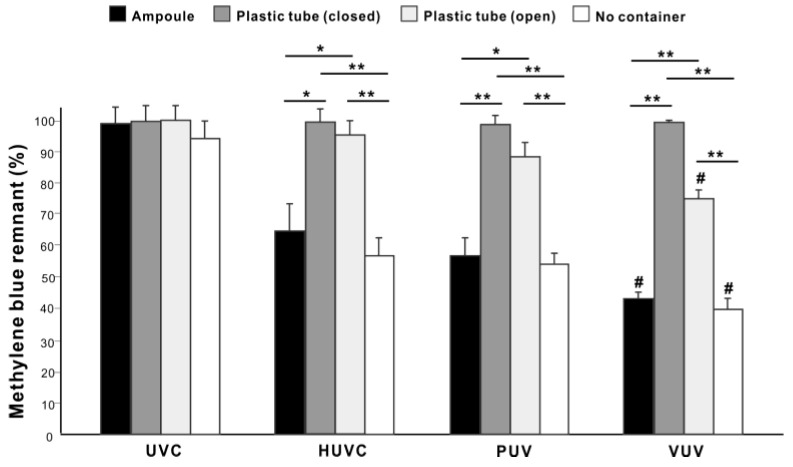
UV-mediated decomposition of methylene blue in different containers. Four different UV light sources were used to treat methylene blue-coated grade 4 titanium specimens for one minute. Quartz ampoules were compared with laboratory-grade plastic tubes with (closed) or without (open) lids. Additionally, direct exposure of UV light was conducted (No container). * *p* < 0.05, ** *p* < 0.01, # *p* < 0.05, statistically different between VUV and other light sources.

We next studied the suitability of quartz ampoules for containing specimens for VUV-mediated organic decomposition. Regardless of the UV light source, there was no significant methylene blue decomposition on titanium specimens placed in laboratory-grade plastic tubes with lids (closed) (Figure 5) but some methylene blue decomposition on titanium specimens in plastic tubes without lids (open), with VUV the most effective UV source (*p* < 0.05; Figure 5). There was significantly more residual methylene blue on specimens treated in open plastic tubes than in quartz ampoules for HUVC, PUV, and VUV (*p* < 0.05). Although there was a trend towards slightly higher organic decomposition on titanium specimens not placed in containers than those in quartz ampoules, the differences were not significant for all UV light sources. Therefore, quartz ampoules do not significantly compromise organic decomposition compared with direct exposure (Figure 5).

### 3.5. Contribution of TiO_2_ Photocatalytic Organic Decomposition

**Figure 6:**
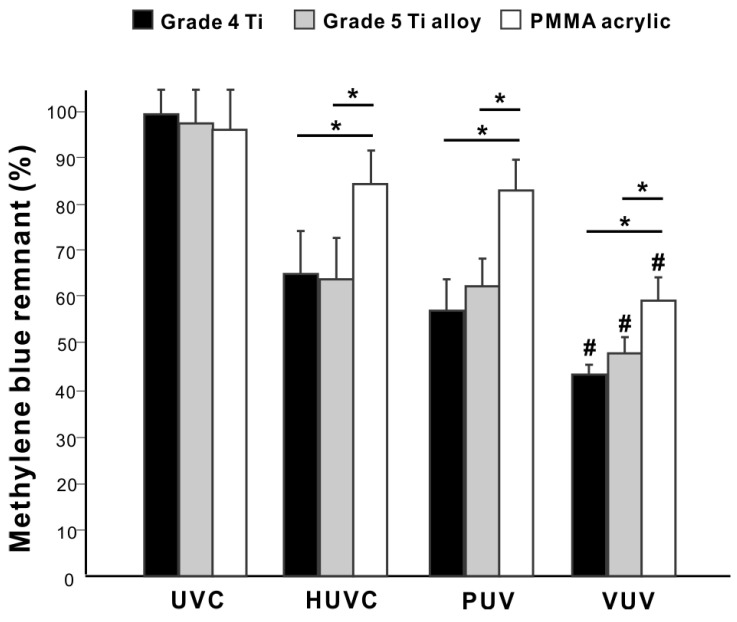
Effects of different materials on UV-mediated organic decomposition. Specimens made from grade 4 titanium, grade 5 titanium alloy, and polymethyl methacrylate (PMMA) resin were compared. A histogram showing remnant methylene blue after one minute of UV treatment (%). * *p* < 0.05, # *p* < 0.05, statistically different between VUV and other light sources.

We wanted to establish whether organic decomposition occurs in the absence of titanium. Methylene blue coating two different titanium (grade 4 titanium and grade 5 titanium alloy) and PMMA specimens were placed in quartz ampoules and treated with different UV light sources for one minute. There were no significant differences in methylene blue decomposition on grade 4 titanium and grade 5 titanium alloy, regardless of the light source (Figure 6). There was significantly more residual methylene blue on PMMA specimens than on grade 4 titanium and grade 5 titanium alloy for all light sources except UVC. There was a 20–25% difference in decomposition between grade 4 titanium and PMMA, indicating a relatively a minor contribution of TiO_2_ photocatalysis to the total organic decomposition.

### 3.6. Different Load-Efficacy Curves of Organic Decomposition between Different UV Light Source

**Figure 7:**
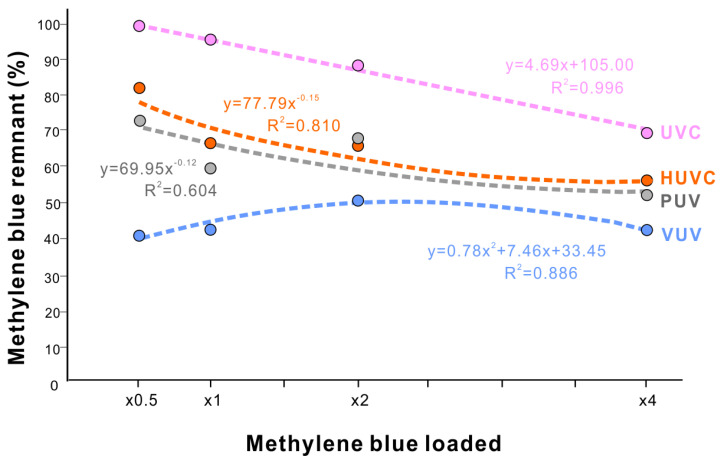
Load testing of organic decomposition by four different UV light sources. Methylene blue at four different concentrations (0.5-times to 4-times the standard concentration used in other experiments) in a quartz ampoule treated with UV light sources for 1 min. Remnant methylene blue is plotted against the methylene blue concentration along with results of the regression analysis. Note that all regression curves move up as methylene blue loading was less except VUV light.

We next examined the capacity of organic decomposition in load-testing experiments for all light sources. We varied the methylene blue density from 0.5-times to 4-times the standard density used in other experiments. At the lowest density (0.5-times), the efficacy of methylene blue decomposition was low for all light sources except VUV, with 73%, 83%, and 100% residual methylene blue for PUV, HUVC, and UVC, respectively (Figure 7). As the density increased, the efficacy increased for PUV, HUVC, and UVC, with their load-residual methylene blue plots fitting a negative exponential curve for PUV and HUVC and a negative linear correlation for UVC. The negative exponential curves for PUV and HUVC indicated that there was a limit/plateau of efficacy with high loads of methylene blue. In contrast, VUV showed a very different, polynomial curve, with low residual methylene blue between 40% and 52% for the loads tested (Figure 7). VUV exerts high responsiveness even for the thinnest coating and consistently high tolerance even for the thickest coating.

### 3.7. Organic Decomposition Corroborated by Hydrophilic Conversion

**Figure 8:**
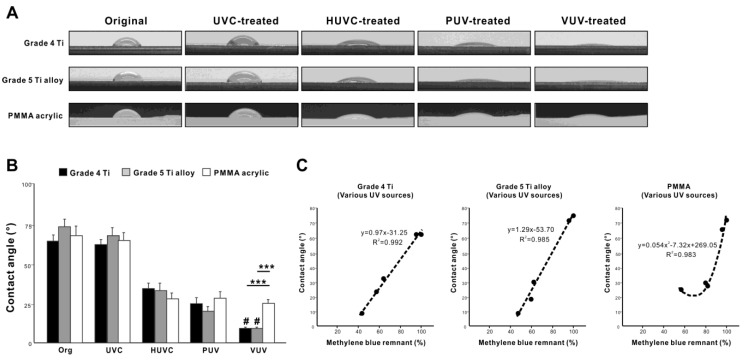
Hydrophilicity/hydrophobicity of the test specimens and its association with organic decomposition. (**A**) Side-view photographs of 3 µL ddH_2_O placed on three different specimens before and after one minute of UV treatment using various UV light sources. (**B**) A histogram showing the measured contact angle based on the images in panel A. *** *p* < 0.001, # *p* < 0.05, statistically different between VUV and other light sources. (**C**) Graphs plotting the contact angles obtained from panel B and residual methylene blue obtained from experiments using four different UV light sources, along with a correlation curve/line and the equations obtained from regression analyses.

We next evaluated the relationship between methylene blue decomposition and hydrophilicity/hydrophobicity of the titanium surfaces, since UV treatment is known to change this surface property. Grade 4 titanium and grade 5 titanium alloy surfaces converted from hydrophobic (defined as a contact angle >45°) to hydrophilic (defined as a contact angle <45°) after one minute of UV treatment, except for UVC, which did not significantly alter the surface wettability (Figure 8A,B). The UV-mediated change in the contact angle was similar between grade 4 titanium and grade 5 titanium alloy, with the greatest hydrophilic conversion observed on VUV-treated specimens with a contact angle <10°. Very interestingly, PMMA surfaces also became hydrophilic after HUVC, PUV, and VUV treatment, although to a lesser degree than grade 4 titanium and grade titanium alloy surfaces (Figure 8A,B).

**Figure 9:**
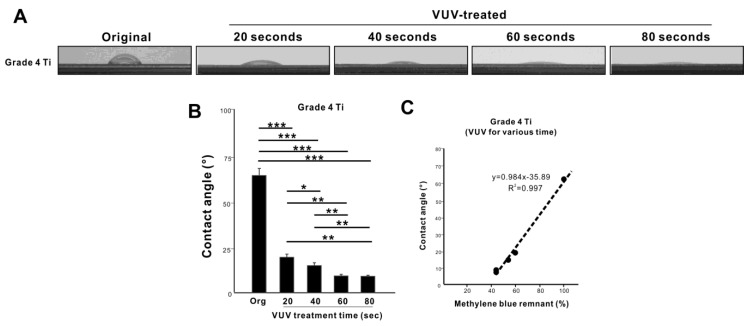
Hydrophilicity/hydrophobicity of grade 4 titanium specimens and its association with organic decomposition. (**A**) Side-view photographs of 3 µL ddH_2_O placed on grade 4 titanium specimens before and after VUV treatment for various treatment times. (**B**) A histogram showing the contact angle measured using the images in panel A. * *p* < 0.05, ** *p* < 0.01, *** *p* < 0.001. (**C**) A graph plotting the contact angles obtained from panel B and residual methylene blue obtained from experiments using VUV (Figure 3), along with correlation lines and equations obtained from regression analyses.

Finally, we plotted residual methylene blue levels against the contact angle for grade 4 titanium, grade 5 titanium alloy, and PMMA specimens (Figure 8C). On grade 4 titanium and grade 5 titanium alloy, the residual methylene blue levels were in near perfect linear correlation with the contact angle with a very high positive coefficient of correlation and a steeper slope of linearity for grade 5 titanium alloy. In contrast, there was a polynomial relationship between residual methylene blue and the contact angle for PMMA surfaces, showing a limit to hydrophilic conversion regardless of methylene blue decomposition. Likewise, we evaluated methylene blue decomposition relative to hydrophilic conversion depending on VUV dose. Grade 4 titanium surfaces became more hydrophilic as the VUV dose increased from 20 to 80 s (Figure 9A,B), with the contact plateauing below 10° after 60 s. Residual methylene blue levels were nearly perfectly correlated with the contact angle (R^2^ = 0.997) (Figure 9C). The slope of the linear correlation was very similar to the one obtained in UV source-dependent experiments on grade 4 titanium (Figure 8C), confirming the accuracy, consistency, and reliability of the correlation.

## 4. Discussion

UV photofunctionalization aims to restore the osteoconductivity of titanium by decarbonizing titanium surfaces and regenerating surface hydrophilicity lost through the natural aging of titanium. Despite the established proof-of-concept and biological effectiveness of photofunctionalization, clinical translation has been challenging. This study revealed that VUV light, with 172 nm peak wavelength, mediates high-efficiency, high-capacity organic decomposition compared with other UV light sources. The positive characteristics of VUV-mediated photofunctionalization pave the way for rapid, efficient, and clinically applicable UV photofunctionalization. VUV achieved maximum organic decomposition after only one minute of treatment, which is clinically acceptable considering currently available UV light source protocols ranging from 12 min to 48 h. Only VUV maintained high methylene blue decomposition in load-testing experiments. Commercially available implants are 40–75% covered with hydrocarbons as an atomic percentage, and the degree of contamination is unpredictable [65,66]. As indicated by the negative exponential curves obtained from load-testing HUVC and PUV, these two UV sources may have a limit their photofunctionalization capacity as the coating becomes denser. In contrast, the polynomial curve for VUV light suggested even greater decomposition efficacy with denser organic coatings. Also, very intriguingly, methylene blue decomposition was less efficient at lower densities for UVC, HUVC, and PUV (Figure 7) but not VUV. The high capacity of VUV to decontaminate materials of carbon regardless of its density is an additional advantage.

VUV was effective when applied to samples in quartz ampoules but not plastic tubes. Given current clinical protocols and the need to avoid bacterial contamination and other physical contact, implant devices must not be exposed nor manipulated prior to placement into patients and should ideally be treated with UV light without first unpacking them from their containers. Impressively, quartz ampoules did not compromise the efficacy of methylene blue decomposition compared with direct treatment of specimens, indicating that most UV light permeated the quartz ampoules [102,103]. However, plastic tubes significantly reduced decomposition efficacy. The higher the energy of UV light, the lower its penetration through the atmosphere and other intervening materials. However, the quartz turned out to be an exception. The performance of a high-energy VUV with its short wavelength was exploitable in synergy with quartz ampoules.

**Figure 10:**
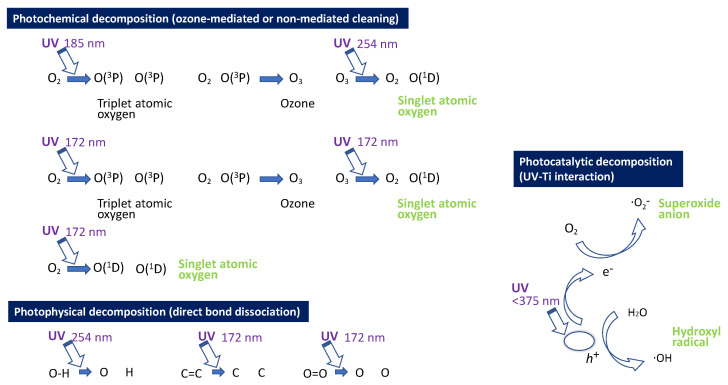
Schematic of three different mechanisms of UV-mediated organic decomposition. During photochemical and photocatalytic decomposition, the generated reactive oxygen species (green highlighted) attack organic molecules. Note that 172 nm VUV light has both photochemical and photophysical advantages, enabling faster and more efficient decomposition.

The rapid and efficient organic decomposition mediated by VUV can be explained by two main UV-mediated carbon molecule decomposition mechanisms: photolytic decomposition and photocatalytic decomposition, the former subdivided into photochemical and photophysical decomposition (Figure 10). Our study showed that, given the rapid and efficient organic decomposition by UV light sources, there is a significant contribution from photolysis. Photocatalysis occurs through interactions between UV light and titanium dioxide, and it is exploited extensively in the environmental sciences and other technologies. The advantage of photocatalysis is that it is triggered by low-energy UV light and applicable to a large surface area. While the effect is slow, it can be continuous throughout the irradiated period, useful, for example, for cleaning rooms and walls. We detected that photocatalytic decomposition contributed 20–25% of the overall effect of HUVC, PUV, and VUV, as shown in the difference between titanium and PMMA specimens (Figure 6), and there was little difference in contribution of photocatalytic decomposition between HUVC, PUV, and VUV sources. We also hypothesized that the high-energy 172 nm VUV rays directly increased the speed and capacity of organic decomposition. As shown in Figure 10, 172 nm VUV light has significant photochemical and photophysical decomposition advantages. A 172 nm UV ray can rapidly produce more reactive oxygen due to exclusive non-ozone-mediated reactive oxygen production, which attacks organic molecules. Furthermore, due to is higher energy, 172 nm UV can directly dissociate stronger atomic bonds than 185 nm UV, resulting in the successful decomposition of a wider range of molecules. The occurrence of both photolytic decomposition mechanisms was confirmed by the substantial organic decomposition even on PMMA specimens and in opaque but open plastic tubes. Again, photolysis contributed 75–80% of the total decomposition after one minute.

Although a previous study demonstrated that there was no topographical change on titanium by UV treatment [80], examining surface properties other than hydrophilicity/hydrophobicity, including but not limited to detail surface morphology and atomic composition, before and after UV treatment is crucial in future studies. This study did not allow for examining these parameters because of the coating of methylene blue and its remnants after UV treatment. Based on the positive result of VUV treatment, future studies will be planned to conduct X-ray photoelectron spectroscopy (XPS) and 3D optical imaging for detail surface chemistry and quantitative roughness evaluation, respectively, potentially influenced by VUV treatment.

We established a new model for UV-mediated organic decomposition on titanium. The assessment of the amount of organic molecules on titanium is a more important determinant of osseointegration than hydrophilicity or hydrophobicity. We used methylene blue as a model organic molecule due to is hydrocarbon-containing molecular structure and clear visualization when coated on materials [104,105]. Methylene blue is also water soluble, which is a necessary property for coating on materials and subsequent photochemical quantification. The degree of carbon contamination of titanium materials varies considerably depending on their age and storage conditions, so it is very difficult to standardize baseline carbon levels. In contrast, the amount of methylene blue coating is very easy to control, as demonstrated in accurate progressive coating in load-testing experiments, which would not be possible without the controlled, artificial contamination used here. The methylene blue model effectively discriminated the degree of organic decomposition between different UV light sources and identified peak decomposition by VUV light, proving the utility, reliability, and sensitivity of the assay. To further establish validity, we compared progressive changes in surface hydrophilicity and methylene blue decomposition during UV treatment (Figure 8 and Figure 9), with the very high correlation between the two variables demonstrating the validity and reliability of the methylene blue model. This model is also simple and cheap to implement so will be useful to screen and test the capability of UV light sources in future studies prior to definitive biological experiments. It will also be important and useful to test the sensitivity and responsiveness of different materials and surfaces, including but not limited to titanium specimens with various surface textures and biomaterials other than titanium such as zirconia.

## 5. Conclusions

Vacuum UV (VUV), emitting 172 nm wavelength light, mediated highly efficient and maximal decomposition of methylene blue on titanium specimens after only one minute. Load-testing revealed consistent decomposition of methylene blue by VUV regardless of the concentration of coating, whereas other UV light sources were less effective with lower density coatings and plateaued capacity with higher density coating. The majority of methylene blue decomposition was through UV photolysis and only 20–25% through UV photocatalysis. Placing a titanium specimen in a quartz ampoule did not compromise methylene blue decomposition, paving the way for treating specimens in their packaging. Our study suggests that VUV now requires further development to quickly and efficiently photofunctionalize titanium implants in in vitro cell culture, pre-clinical, and clinical settings.

## Data Availability

Data availability on request from the authors.
